# Advances in Nano-Electrochemical Materials and Devices

**DOI:** 10.3390/nano14080712

**Published:** 2024-04-18

**Authors:** Mei Wang, Xuyuan Chen, Nabin Aryal

**Affiliations:** 1 State Key Laboratory of Quantum Optics and Quantum Optics Devices, Institute of Laser Spectroscopy, Collaborative Innovation Center of Extreme Optics, Shanxi University, Taiyuan 030006, China; 2 Department of Process, Energy and Environmental Technology, Porsgrunn Campus, University of South-Eastern Norway, 3918 Porsgrunn, Norway

Nano-electrochemical materials and devices are at the frontier of research and development, advancing electrochemistry and its applications in energy storage, sensing, electrochemical processing, etc. The synergy of nanotechnology and electrochemistry has led to advances in nanostructured electrode materials [1,2]. Nanotechnology is supported to advance nanomaterials with high spatial surface area, nanosized and porous-induced physical effects, and multi-dimensional structure construction, which boosts prominent properties bordering its potential applications in developing electrochemical devices.

The recent critical developments in nano-electrochemical materials are devices that focus on developing nanostructured electrode material [3], nano-electrochemical sensors [4], nanomaterials for energy storage and conversion [5,6], etc. Developing a novel material synthesis process enables smaller-scale prototypes for application, optimization, and innovation experimentation. Therefore, researchers must explore novel concepts and apply synergy from multiple disciplines to explore fundamental science. That could build a strong foundation for further industrial applications, technology upscaling and commercialization. The research and development on laboratory-scale applications required controlled environmental research with various parameters to bridge the gap between concepts and laboratory-scale innovation testing. In this Special Issue, various advanced nano-scale materials and novel processing techniques are reported, including material synthesis, structure design, interface engineering, and characterization methods.

Demonstrating and developing new nanomaterials and devices are not only academic exercises for teaching but also include heavy practical applications. However, practical technical information on the innovation and application of electrochemical materials and devices is still limited. To overcome such a gap, this Special Issue was aimed at providing technical details on nano-electrochemical materials and devices. The Special Issue was focused on summarizing the advances in the design, development, manufacture, and application of nano-electrochemical materials and devices. In the Special Issue, recent advances in nanomaterial development, particularly graphene-based, polypyrrole-based, and carbon tube-based material developments, were critically reviewed. The critical review summarized the applications of carbon-based materials in electrochemical sensor device applications, photocatalyst and electrocatalyst applications, and biosensors for early disease diagnosis. Experts wrote the individual scientific manuscripts published in this Special Issue, consolidating the most recent state-of-the-art and focusing on innovation.

In the field of energy conversion and storage, novel electrode material synthesis, especially N, S, and Co-doped graphene (Contribution 1), and constructing FeNi_3_/C nanorods (Contribution 2) to enhance oxygen reduction reactions have been tested. A low-loaded silver-based electrode is also fabricated via the sputtering deposition technique for CO_2_-to-CO conversion directly from the capture medium (Contribution 3). Moreover, solid-state batteries have boosted the development of solid-state electrolytes. In this Special Issue, the electrochemical performances of PVDF-HFP-LiClO_4_-Li_6.4_La_3.0_Zr_1.4_Ta_0.6_O_12_ composite solid-state electrolytes were researched, which is essential for the development of high-energy density all-solid-state lithium-ion batteries (Contribution 4). A three-matrix solid electrolyte membrane in air was also tested and published (Contribution 5). The critical scientific information from this research could be an essential reference for further developing all-solid-state batteries. In addition, solid-state lithium-sulfur batteries have recently received widespread attention for energy storage due to their high current density, performance, and economics [7]. Therefore, composite polymer electrolytes and nitrogen-doped porous carbon fiber composite cathodes were synthesized (Contribution 6), and their electrochemical performance was tested to advance the technology. Advances in the separator also promote improving the performance of lithium-sulfur batteries. An aramid fiber-modulated polyethylene separator was reported as an efficient polysulfide barrier for high-performance lithium-sulfur batteries, providing a facile way to fabricate the separator for inhibiting polysulfides in the lithium-sulfur battery (Contribution 7).

Nowadays, due to the increasingly severe greenhouse effect, the treatment and utilization of carbon dioxide (CO_2_), which is the biggest contributor to greenhouse gases (GHGs), has become a worldwide hot topic. Current utilization methods for CO_2_ include chemical conversion, photoreduction, electrochemical reduction [8], bioconversion, etc. In this Special Issue, two review papers were published, summarizing recent progress in photoreduction and electrochemical reduction of CO_2_. One paper reviewed the carbon tube-based cathode for Li-CO_2_ batteries (Contribution 8). As an electrochemical device that can capture, fix, and convert CO_2_, electric energy can be stored for energy utilization in various applications. The other review paper reviewed the multifunctional graphene-based nanocomposites for photocatalysis and electrocatalysis applications, including photocatalytic hydrolysis, pollutant degradation, and the photocatalytic reduction of CO_2_ (Contribution 9).

Recent laboratory research has shown that metal-organic framework (MOF) materials have demonstrated superior performances because of their high-tunable conductivity and their structure’s pore size [9]. One of the research papers published in this Special Issue is the synthesis of 4,4′-biphenyl dicarboxylic acid-based nickel metal-organic frameworks for supercapacitor applications (Contribution 10). The research evidence published in the manuscript will be beneficial as reference information to develop high-performance hybrid MOF composites for future electrochemical energy storage applications.

Employing electrochemical devices as sensors represents a groundbreaking capability in identifying environmental traces, significantly contributing to the monitoring and restoration of natural ecosystems. This sensor technology has also propelled advancements in medical diagnostics and industrial processes. Apart from electrochemical devices, nanomaterials have also gained broader application in medical applications for disease detection. Towards such aims, one of the manuscripts developed a non-enzymatic electrochemical sensor for the detection of glutamate based on an advanced porous carbon electrode, and impressively, a novel detection mechanism based on Cu ions was first published (Contribution 11). Recently, conductive polymers have also drawn great attention in the field of electrochemical sensors. A review of polypyrrole-based electrochemical biosensors for the early diagnosis of colorectal cancer is published, reporting the properties, synthesis techniques, and applications of the biosensors (Contribution 12). These achievements demonstrated the advances and innovation of electrochemical devices for medical and biological applications, such as microbial fuel cells and amicrobial electrochemical systems.

In conclusion, nanometer-scale electrochemical materials and devices have made gratifying progress in various fields in recent years. This Special Issue reported several novel research works on advanced nanomaterials and nano-systems for oxygen reduction reactions, batteries, supercapacitors, and sensors. Recent achievements in photocatalysis and electrocatalysis, biosensors, and Li-CO_2_ batteries were comprehensively summarized in three review papers. We strongly believe that this issue will draw wide attention in the field of electrochemical materials and devices and promote the development of advanced functional nanomaterials and nanotechnologies.
